# Open and reproducible science practices in psychoneuroendocrinology: Opportunities to foster scientific progress

**DOI:** 10.1016/j.cpnec.2022.100144

**Published:** 2022-05-30

**Authors:** Maria Meier, Tina B. Lonsdorf, Sonia J. Lupien, Tobias Stalder, Sebastian Laufer, Maurizio Sicorello, Roman Linz, Lara M.C. Puhlmann

**Affiliations:** a Neuropsychology, University of Konstanz, Germany; b Institute for Systems Neuroscience, University Medical Center Hamburg-Eppendorf, Hamburg, Germany; c Centre for Studies on Human Stress, Department of Psychiatry and Addictology, University of Montreal, Québec, Canada; d Clinical Psychology and Psychotherapy, University of Siegen, Germany; e Division of Clinical Psychological Intervention, Department of Education and Psychology, Freie Universität Berlin, Berlin, Germany; f Department of Psychosomatic Medicine and Psychotherapy, Central Institute of Mental Health, Heidelberg University, Germany; g Max Planck Institute for Human Cognitive and Brain Sciences, Leipzig, Germany; h Leibniz Institute for Resilience Research (LIR), Mainz, Germany

**Keywords:** Psychoneuroendocrinology, Open science, Reproducibility, Preregistration, Multiverse, Registered report

## Abstract

This perspective article was written by invitation of the editors in chief as a summary and extension of the symposium entitled *Psychoneuroendocrine Research in the Era of the Replication Crisis* which was held at the virtual meeting of the *International Society of Psychoneuroendocrinology* 2021. It highlights the opportunities presented by the application of open and reproducible scientific practices in psychoneuroendocrinology (PNE), an interdisciplinary field at the intersection of psychology, endocrinology, immunology, neurology, and psychiatry. It conveys an introduction to the topics preregistration, registered reports, quantifying the impact of equally-well justifiable analysis decisions, and open data and scripts, while emphasizing ‘selfish’ reasons to adopt such practices as individual researcher. Complementary to the call for adoption of open science practices, we highlight the need for methodological best practice guidelines in the field of PNE, which could further contribute to enhancing replicability of results. We propose concrete steps for future actions and provide links to additional resources for those interested in adopting open and reproducible science practices in future studies.

## Highlights

• Open science practices (OSPs) are not yet regularly applied in Psychoneuroendocrinology (PNE).
• We summarize opportunities, challenges, and ‘selfish’ reasons to adopt OSPs in future PNE studies.
• We discuss their potential to enhance data quality, transparency, and progress in the field of PNE.
• We provide introductory resources for researchers aiming to adopt them.
• We encourage the development of methodological best practice guidelines and self-correction mechanisms in PNE.

## 1 Introduction

Open science reflects the idea that “scientific knowledge of all kinds, where appropriate, should be openly accessible, transparent, rigorous, reproducible, replicable, accumulative, and inclusive” [1] (see → Glossary). It thus makes the scientific process more transparent and constitutes one way to ensure that results are replicable and robust. This is important because unreliable findings prevent scientific progress [2]. Nevertheless, open science practices are not applied widely in the field of psychoneuroendocrinology (PNE) to date. While they certainly do not constitute the only approach that increases reliability of results, there lies great potential in the adoption of open science practices for individual researchers, as well as the whole field of PNE. The aim of the current article is therefore to showcase examples of open science practices that can be adopted more comprehensively in the field of PNE on an individual as well as structural level. We discuss challenges and opportunities of such practices in our field and propose concrete steps for future actions. Further, we provide a collection of resources to support the implementation of open science practices for individual researchers in PNE.

## 2 Replicability in the field of psychoneuroendocrinology

The use of open science practices has gained considerable popularity as a direct response to the finding that many published studies – in some fields indeed the majority of studies – cannot be replicated (often referred to as the so-called ‘replication crisis’; [3]. There are several factors that contribute to low replicability of results. First, there is a strong and consistent ‘publication bias’ [4,5] towards publishing novel, and statistically significant results, while non-significant findings often remain unpublished (‚file drawer problem‘; [6,7]. Second, this at large originates from the current incentive structures (‘publish or perish’; [8]. These structures promote questionable research practices (QRP) such as modifying the statistical procedure until a significant result is obtained (p-hacking [[9], [10], [11], [12]]; for a simulation that demonstrates effects of p-hacking see https://shiny.psy.lmu.de/felix/ShinyPHack/), generating the Hypothesis After the Results are Known (HARKing; [13], and/or selectively reporting significant analyses (cherry picking; [14]. Third, even if analyses are sound, methods and analysis pathways are often not transparently or comprehensively reported [15], which can lead to a robustness problem (i.e. different analysts produce different results when answering the same research question and using the same dataset; [16]). Fourth, the totality of measures used (questionnaires, experimental manipulations, etc.) may not be reported. Open science addresses these issues, which is why individual researchers as well as entire fields of science can ultimately benefit from adopting open science practices.

By now, several open and reproducible science practices have been discussed to enhance reproducibility (see → Glossary) and transparency in research. Some involve improved detection of statistical errors and inconsistencies (e.g. http://statcheck.io, [17]; GRIM; [[18], [19], [20]]. The majority, however, focuses on aligning the research process and publication culture to optimize scientific progress. Following the failure to replicate a significant proportion of studies in the field of psychology, Nosek and colleagues (2012) proposed various structural remedies in order to increase the quality of published research, including pre-specifying hypotheses and analysis plans (preregistration and registered reports; see → Glossary; [21], publishing null findings [22], sharing analysis code and data [23], and testing the robustness of results across analytical approaches [16,24]. In sum, these practices aim to maximize transparency, accessibility, and reliability of research.

Like other fields of research, replicability challenges, potential reasons and solutions have been discussed in PNE. One cautionary example is provided by oxytocin administration research: while initial studies showed promising findings of increased trust and therefore sparked interest in clinical application (cf. [[25], [26], [27]], these results were likely pushed by publication bias [28] and do not replicate well [29,30]. Reasons for this may include a shaky basis of hypotheses [31], but also unresolved questions on how to determine oxytocin reliably using biochemical assays in the first place [32]. Using this example, it becomes evident that besides the above-mentioned generic factors, there are also field-specific factors that contribute to low robustness of results, which we want to highlight in this piece.

In PNE, there are currently only very few consensus guidelines [33] on best methodological practices to obtain specific biomarkers [34]. Simultaneously, endocrine assessments have become more and more accessible in the last decades. Many hormonal measurements are easy to implement at relatively low cost (e.g., measuring hormones non-invasively in fluids and tissues other than blood).Adding the measurement of hormones to a research design without the corresponding methodological knowledge or *a priori* hypotheses has consequently become easier (fishing net approaches). Hence, it is critical to ensure that future work in the field of PNE follows good methodological and statistical practices.

There are already first attempts and examples of open and reproducible science practices in PNE, for example preregistered studies (e.g. Refs. [35,36], or registered reports (e.g., Ref. [37]. Also, P-curve analyses that aim to estimate whether published results provide evidence for a true underlying effect have been published (e.g., Ref. [38]. However, to date, open science practices are not applied widely in the field of PNE. A screening of all articles published in the journals *Comprehensive Psychoneuroendocrinology* and *Psychoneuroendocrinology* in 2021 showed that 9% of studies in *Comprehensive Psychoneuroendocrinology,* and 7% of studies in *Psychoneuroendocrinology* were preregistered. In contrast, numbers can rise to 30–50% in psychological journals that explicitly encourage preregistration [39]. A similar picture emerges when looking at the practice of sharing analysis scripts (Comprehensive Psychoneuroendocrinology: 4%, and Psychoneuroendocrinology: 6%) or data (Comprehensive Psychoneuroendocrinology: 2%, and Psychoneuroendocrinology: 7%; for more details on the methodological approach to obtain these percentages and the raw data see → supplementary information). While these two journals represent only a fraction of the publications in our field, the numbers nevertheless suggest that PNE can still improve in this area.

An overall conceptual and structural change is needed to facilitate and foster long-lasting developments towards open and reproducible science in our field. There is a lot to gain from following the lead of other fields as well as the increasing demand by funders (NIH has made open data mandatory as of 2023 [40]; and other stakeholders: Adopting open and reproducible science practices can enhance overall credibility of research fields. Next to the organizational/structural level, individual researchers can contribute to this change by implementing open science practices routinely in their studies. We are aware that the implementation of such practices comes at a certain cost, as learning new skills and implementing new methods takes time [41,42]. Yet, we are convinced that open science practices are an important tool that can foster scientific progress [2] and that can offer advantages for individual researchers. Given the close ties of PNE to healthcare applications, high replicability might even indirectly prevent dangers to human health and welfare (e.g., Ref. [43].

## 3 Approaches to enhance reproducibility and why they are relevant in PNE

There are many ways to improve reproducibility and transparency in science. In PNE, papers discussing problems and generating consensus on the best methods to obtain reliable hormone samples and analyses have been published [33,34] and the development of new guidelines will continue to be important. Here, we showcase four additional approaches that were presented in the symposium entitled *Psychoneuroendocrine Research in the Era of the Replication Crisis* at the virtual conference of the *International Society of Psychoneuroendocrinology* 2021. Specifically, we discuss how the implementation of (1) **preregistration,** (2) **registered reports,** (3) quantifying the impact of (the multitude of equally-well justifiable) analysis decisions (e.g., by using specification curve, **multiverse analysis**), and (4) public sharing of **data and analysis code** could be first steps to enhance reproducibility of findings in the field of PNE.

### 3.1 Preregistration

Preregistration is probably the best-known [21] and the most widely adopted open science practice [44]. It involves pre-specifying one's hypotheses and statistical approach prior to data collection or analysis in a time-stamped document. As such, it clearly separates predefined hypothesis-testing (confirmatory) from more liberal hypothesis-generating (exploratory) research (see → Glossary, [21,45]. Preregistering one's work can therefore be a nudge to critically reflect the planned study design and analytical strategy*.* Following publication, preregistration can greatly enhance the credibility of the eventual result [46]. It serves as proof of which hypotheses and analyses were planned truly *a priori* and accordingly chosen to be reported independent of their statistical significance. In studies that may influence treatment choices or health outcomes, such as clinical trials, preregistration has therefore been a requirement for some time (e.g., Refs. [47,48]. While PNE researchers often address preclinical questions, this work ideally cumulates in advances in healthcare such as new treatment approaches. A current example is the development of neuroinflammation as a new biomarker and target for depression treatment [49], which was influenced by the discovery of stress-induced release of inflammatory proteins and cytokines during laboratory tasks [50,51] such as the Trier Social Stress Test (TSST; [52].

Planning and committing to a detailed methodological and analytical research plan *a priori* can be challenging. In the case of PNE, this task may even be aggravated by the typically complex data that are subject to various potential confounding variables such as menstrual cycle phase [53], or circadian rhythms [54]. For this reason, scientists may shy away from preregistration or assume that any kind of deviation from the original plan may lead to the invalidation of their preregistration. It is, however, important to note that preregistration is primarily about transparency; it is ‘a plan, not a prison’ [21]. It is indeed not uncommon that researchers need to deviate from data collection and analysis plans (e.g., when assumptions for statistical tests are not met). As long as deviations are well-justified and transparently reported, such as through preregistration amendments (https://help.osf.io/article/110-introduction-to-updating), they do not undermine the benefit of preregistration [21].

In addition to determining an analysis plan, another important part of preregistration is establishing a target sample size prior to data collection, for example, based on power calculations that build on effect sizes reported in the literature [55]. This prevents additional data from being collected if the specified hypotheses are not confirmed. Transparently reporting effect sizes to guide power calculations is a challenge that needs to be addressed by the whole field, but can eventually ease preregistration and promote a sustainable use of resources. While preregistration was originally devised for newly collected, unobserved data, a planned analysis of existing or previously observed data – a so-called secondary data analysis (see → Glossary) – can also be preregistered, and some even argue that it is particularly valuable in this case [56].

In wake of the replication crisis, preregistration has increasingly become important and adopted widely, e.g. in the field of psychology [44]. As the numbers of our paper screening suggest, there is still some potential to adopt preregistration more regularly in PNE studies. With all its challenges, preregistration can actually be as simple as answering 10 questions (cf. https://aspredicted.org). Nonetheless, it should be noted that a more detailed preregistrations are better suited to prespecify the study and analysis plan [57]. Fortunately, there are many tutorials and resources to help you get started with preregistration (for resources see e.g., → Box 1).

Box 1 **Resources for first steps towards open and reproducible science practices.**

**Table:** 

	overview articles, e.g., on benefits and challenges	tutorials, templates, and open-source code
*open science practices and preregistration*	open science practices, see [58] preregistration, see [44,46,59] secondary data analysis [56]:	template collections: https://prereg-psych.org/index.php/rrp/templates or https://osf.io/zab38/wiki/home/
*registered reports*	see [[60], [61], [62]]	template collection: https://osf.io/93znh/
*multiverse analysis*	see [24,63]	coding tutorial: https://www.youtube.com/watch?v=udG-5DiJJ4w R packages: Multiverse (see, https://cran.r-project.org/web/packages/multiverse/readme/README.html) or mverse (see, https://mverseanalysis.github.io/mverse/) or specr (see https://cran.r-project.org/web/packages/specr/vignettes/specr.html)
*transparency (open data, open code)*	see [64]; or https://psych-transparency-guide.uni-koeln.de respectively	synthetic data, see [65,66]

Alt-text: Box 1

Taken together, we encourage readers to contribute to the change towards more open and reproducible PNE with their own preregistrations.

### 3.2 Registered reports

As an alternative to preregistration, researchers may consider to conduct and publish their study as a registered report (RR; see → Glossary; [67]. While preregistrations are a study add-on, RRs are a publication format of their own that involves a unique two-stage review process (see https://www.cos.io/initiatives/registered-reports). Like preregistrations, RRs require a detailed methodological and analytical *a priori* plan. Researchers start an RR by submitting the introduction and methods part of their future manuscript as a research proposal to one of the journals offering this publication format (cf. https://www.cos.io/initiatives/registered-reports, section ‘participating journals’; last accessed on March 11, 2021). An initial peer review evaluates the proposed study's rationale, theoretical justification and methods before the study is conducted. After potential revisions, a positive evaluation of the proposed plan ensures in-principle acceptance (IPA) of the manuscript for publication [68]. Subsequently, data are collected and analyzed, and the manuscript including results and discussion is subjected to a second peer review. At this stage, adherence to the prespecified analytic protocol and presentation as well as interpretation of results is valued – rather than the significance of results, novelty, and the confirmation of hypotheses per se.

This two-stage process effectively eliminates publication bias towards significant findings and null-results [41,69,70]. Registration of a peer-reviewed analytical plan also effectively forestalls QRPs like p-hacking and ensures transparent reporting of methods and analysis pathways. Consequently, conducting a study as a RR can greatly enhance its credibility. Critically, the benefits of peer-review are maximized when – at the initial proposal stage – study design and analytical approach can still be adjusted based on the feedback (‘feedback before action’, https://osf.io/93znh/last accessed on March 28, 2021). Indeed, first studies attempting to assess the quality of RRs suggest that their methodological and overall quality is perceived as higher as compared to standard publications [71,72]. Enhanced computational reproducibility of results in RRs has also been noted, linked to the strong encouragement and higher rates of data and code sharing in this format (cf [73]. At the same time, no disadvantages in terms of article impact have been observed [74]. For the individual researcher, the RR format can thus serve as a signpost of study quality, while the IPA presents a strong safety asset that provides a clear roadmap irrespective of study outcomes.

In the realm of PNE, hormonal measures are effortful to collect and subject to various confounding variables. Therefore, additional expert input during the conception phase of a study could directly impact data quality. During the traditional peer-review process, reviewers can only raise concerns about non-adherence to recommendations and best-practice guidelines *after* data collection. In PNE, a recent analysis showed that while expert consensus guidelines for the reliable assessment of the cortisol awakening response [33] are regularly cited in articles published in *Psychoneuroendocrinology*, only a minority of studies actually follow the most critical guidelines (Stalder et al., in prep). In RRs, reviewers can detect deviations from current best practice approaches *prior* to data collection, when recommendations can still be implemented. Therefore, RRs allow for the enforcement of methodological rigor at an earlier stage as usual.

Despite of these advantages, to date, examples of RRs in PNE (e.g. Refs. [37,75], are still rare, and we are not aware of PNE specific journals that offer the publication of RRs (cf. https://www.cos.io/initiatives/registered-reports section ‘participating journals’, last accessed on March 11, 2021). This might hinder PNE researchers to opt for this format, as the publication process of RRs remains unfamiliar and might seem to entail additional expenses (e.g., waiting for and incorporating reviewers' feedback instead of starting with data collection immediately).

One of the most frequently expressed concerns regarding RRs (and preregistration in general, e.g. Ref. [76], is that they would limit exploration and thus stifle discovery. While RRs mainly pertain to confirmatory research, additional exploratory analyses are possible and not at odds with the format (some researchers even call for preregistration of exploratory research questions; [77]. Rather than precluding exploration, researchers and readers are pushed to reflect on the confirmatory/exploratory intentions of their research while not conflating the (statistical) tools for these complementary approaches [60,78]. Like preregistrations, RR protocols are not prisons and deviations are possible even after receiving IPA, provided they are justified and transparently reported. Deviations should be clearly labelled during secondary peer review and, in case of uncertainty, may be discussed with the editors beforehand.

Overall, preregistration and RRs are effective tools to improve the quality and credibility of studies, journals, and eventually, a research field [44]. Similar to clinical trial registrations, they forestall harmful practices that may bias results, such as selective reporting and HARKing, which encourages the transparency necessary for a field with close ties to medical research like PNE. At the same time, these tight links to clinically relevant application necessitate a particular strong body of replications and confirmatory research, which RRs and preregistration facilitate. We therefore encourage broader adoption of RR by PNE researchers and journals to facilitate sustainable scientific progress. For more detailed resources that can serve as a starting point for interested readers see → Box 1.

### 3.3 Multiverse-type of analyses

While preregistration and RRs are helpful in increasing transparency (e.g. Ref. [21], and reducing publication bias, they do typically leave one potential challenge for replicability unaddressed: the co-existence of multiple equally reasonable data recording, processing and/or analysis pipelines (for demonstration see for example https://r.tquant.eu/KULeuven/Multiverse/). The multitude of different approaches becomes evident for instance in multi-analyst studies, in which the same dataset is analyzed independently by different researchers [16], or through systematic literature searches [[79], [80], [81]].

In the PNE literature for example, cortisol stress reactivity is operationalized as a change score, the area under the response curve, or a time trend. Nevertheless, even within one study, results are not often consistent across these operationalizations (e.g., Refs. [36,82,83]. Similar methodological heterogeneity is found for the definition of cortisol stress responders and the handling of these, even though definitions have been published in the past [84]. As a third example, processing steps to deal with normality violations in repeated endocrine data vary greatly, although there are data-driven methodological recommendations [85]. Unless compared directly and empirically, it is impossible to quantify the impact of such methodological heterogeneity on results and replicability.

As a potential solution to this, the ‘multiverse approach’ (i.e., systematic robustness analyses) has recently been suggested [24] which is used to investigate if and how each of these in principle equally justifiable decisions impacts on the results: In data multiverse analyses, the same raw data is processed into a multiverse of processed datasets (each referred to as a ‘universe’) depending on different data acquisition and/or processing choices – all potentially equally reasonable in light of the absence of empirical and/or theoretical criteria to guide the researchers' decisions. This data multiverse (i.e., the sum of all universes) inevitably implies a multiverse of statistical results given a single set of identical raw data and identical statistical models applied [16,24] and can inform on the robustness of the effect of interest against alternative processing pathways. Further, it can aid the development of empirically based standardized processing pipelines. To this end, multiverse-type of analyses can provide many of the answers that many-analyst approaches can deliver (e.g. Refs. [16,86], with less effort and resource investment. Similar concepts such as model multiverse [24] and design multiverse [87] target heterogeneity in statistical approaches and experimental design specifications, respectively.

The selection and justification of to be included specifications is key in multiverse-type of studies – in particular as the ‘full’ multiverse consists of an infinite number of options covering data acquisition, processing and analytical decisions [63]. In fact, it can often be advantageous to focus on ‘a’ multiverse with a more limited set of decision nodes – sometimes referred to as ‘manyverse’ as a first step.

Multiverse-type of analyses are computationally challenging even though a number of helpful tools have been developed to facilitate these types of analyses (for resources see → Box 1) and to illustrate the results more comprehensively and more detailed through specification curve plots in concert with specification cure analysis [39] than through a histogram of p-values [24]. First attempts to explore multiverses have already been made in the field of PNE (e.g. Refs. [88,89], and we encourage readers to make use of such analyses to complement open science practices and quantify the impact of equally-well justifiable analysis decisions. As a result, these analyses can guide recommendations and best practice guidelines and have strong potential to directly contribute to the aim of enhancing reproducibility in our field.

### 3.4 Open data

While preregistration and RRs, as well as multiverse analyses can enhance credibility of results, only making research materials, data, and code available to the public can ultimately allow other researchers to reproduce the analyses and results exactly. In fact, publishing these data openly is increasingly being advocated by stakeholders, politics, scientific societies [90], funders, journals as well as researchers. The benefits of publicly available data have been outlined in several excellent sources [64,91,92]. Furthermore, data sharing can even benefit researchers' careers through catalyzing new collaborations [93]. Key advantages of open data thus include the sustainable use of scarce resources (manpower, time and funds), the acceleration of scientific progress (as evident from the case of open data during the COVID-19 pandemic [94], enhanced reproducibility as well as facilitated cumulation of scientific knowledge.

To this end, open data facilitates data aggregation across studies and labs and can thereby further foster theory development and refinement. Some examples in PNE include the EU stress database (https://stressdatabase.eu/ [95]; and the Ulm TSST-database [96]. Such mega-analysis approaches, however, also highlight the importance of standardized data formats and metadata. The benefits of open data can only be realized when data is shared in a findable, accessible, interoperable and reusable way (cf. FAIR principles, https://www.go-fair.org/fair-principles/) and with adequate licenses (cf. https://creativecommons.org). These challenges related to publicly sharing data and re-using publicly available data for re-analysis have been discussed previously in biological psychological and (cognitive) neuroscience [97]. In general, a detailed codebook and, ideally, open code are important to allow re-users to exactly understand how data were assessed and processed, as procedures are often reported only in brief in publications. To support replicability, fully automated data processing pipelines have been developed in a number of research areas, though not yet in the PNE field [98]. Many of these practical and ethical challenges and considerations [99] are field-specific (cf. brain imaging data structure [BIDS] for functional neuroimaging data [100], and we highlight the need for a PNE specific discussion on data and code sharing, especially as the publication of certain raw biomarker material can threaten the anonymity of participants. In certain cases, raw data simply cannot be shared, because they contain sensitive clinical information. This is often the case in PNE. However, accessible tools exist to help create *synthetic datasets*, which protect participants’ identity while allowing data sharing to facilitate reproducibility and aggregation [65,66].

Although the field as a whole is needed to develop data sharing recommendations and guidelines, we want to encourage individual researchers to make their materials, data, and analysis codes available on public repositories to allow for reproducibility of their results. There are several resources that can be helpful in this context (see e.g., → Box 1) and we hope that more PNE specific guidelines will be developed in the future.

## 4 Prerequisites and next steps

The above-described approaches provide steps for increasing the transparency of research, enabling the replication of results, promoting the sustainable use of resources, and overall increasing the knowledge cumulation of our research field. By extending our efforts towards realizing some of these strategies in future research (see → Box 2), we hold great potential for advancing overall progress and countering concerns about reproducibility and replicability, so that PNE can fully exploit its potential by adopting new scientific practices. We, therefore, hope to motivate researchers to implement open science practices in their own studies in the future.

Box 2 **Where to go from here?**

**Table:** 

Actor	Proposed actions to foster reproducibility
Scientific societies	*Immediately implementable* ● explicitly encourage the implementation of open science practices through public statements to the members ● raise awareness through dedicated symposia or panel discussions at conferences and meetings ● offer workshops and trainings on open science practices and PNE specific methodology ● sign the Declaration of Research Assessment (DORA) statement (https://sfdora.org) *Implementable on medium term* ● form open science task forces and task forces on methodological questions ● explicitly reward open and reproducible science practices, e.g., through specific prizes *Implementable on long term* ● create spaces for and encourage team science and multi-analyst projects, e.g., by founding grants that fund collaborative, open science projects
Individual researchers	*Immediately implementable* ● take part in workshops and trainings on open science ● adopt open science practices in own research ● value open science practices in the work of others, e.g., as a reviewer ● encourage transparency as a reviewer (e.g., by using the Standard Reviewer Statement for Disclosure of Sample, Conditions, Measures, and Exclusions, https://osf.io/hadz3/) *Implementable on medium term* ● join open science task forces ● educate own students and co-workers *Implementable on long term* ● consider open science practices in grant applications, committee work, etc.
PNE as a field	*Immediately implementable* ● collaboratively develop best practice guidelines, e.g., on how to use and report methods and procedures ● collaboratively develop harmonization of processing and analysis steps *Implementable on medium term* ● kick-off multi-center studies/many analysts studies ● start data pooling projects that are openly available *Implementable on long term* ● form consortia that regularly update and expand best practice guidelines based on new developments in the field
Journals	*Immediately implementable* ● actively encourage and value open science practices such as preregistrations, e.g., through open science batches (e.g., https://osf.io/6q7nm/) ● publish null findings and (independent) replication attempts ● commit to TOP guidelines (https://osf.io/9f6gx/wiki/home/) ● offer open access waivers for best practice guidelines to ensure availability to whole field ● make data and code sharing statements compulsory *Implementable on medium term* ● ensure rigor methodology via author guidelines and implement checks of minimal requirement standards for publication in accordance with best-practice guidelines ● implement reproducibility checklist in peer review process ● regularly call for special issues focusing on methodological questions ● implement registered report format *Implementable on long term* ● switch to open access model ● make data and code sharing compulsory (if data sensitivity does not prohibit)
Teachers	*Immediately implementable* ● include the topics of open science and reproducibility into the standard curriculum (for materials, see https://forrt.org/or https://www.osc.uni-muenchen.de/toolbox/resources_for_teaching/index.html) *Implementable on medium and long term* ● regularly offer open science workshops and trainings

Alt-text: Box 2

To increase the implementation of open science practices, like preregistration and RRs, changes on several levels may be beneficial, including on the levels of scientific societies, journals [101], and teaching staff [[102], [103], [104]]. First, *scientific societies* bear a great responsibility for promoting as well as representing a scientific field to the world. In this context, the research standards which a society imposes on itself, the importance it assigns to topics, such as methodological quality and transparency, and self-correction measures to retain credibility reflect on the scientific field as a whole [105]. In our opinion the issues arising from low replicability warrant a clear statement that goes beyond lip service. This may entail things like the specific recognition of open science initiatives in the field, the organization of panel discussions on the topic at conferences, the encouragement to form open science task forces within the society, the provision of open science workshops, but also signing the Declaration of Research Assessment (DORA) statement (https://sfdora.org).

Second, *journals* and editors in PNE may take steps to actively encourage open science practices, like preregistration and data sharing. A strong sign of endorsement would further be the possibility to hand in proposals for RRs. Hence, a clear commitment in the author guidelines to both approaches is in our opinion a good start. Building on this, editors and journals could consider adding preregistration as a minimum requirement for publication. To enable a smooth transition, a stepwise implementation of this requirement is conceivable (i.e., a strong recommendation in the guide for authors for X years followed by a binding requirement after the transition period).

These endorsements could be complemented by considering creating journal specific open science statements based on an individualized adoption of the Transparency and Openness Promotion Guidelines (cf. TOP guidelines, https://osf.io/9f6gx/wiki/home/). This would further incentivize researchers to increase their efforts to follow open science practices and provide clear reporting guidelines. In this context, journals could take further steps to implement simple reviews of open science practices in their review process, e.g., by including a reproducibility checklist in their peer-review form [101].

Lastly, journals may also demonstrate their commitment to increase replicability by creating a ‘replication study’ article type, or regular special issues devoted to replication studies. This could greatly benefit PNE as replicability is estimated to still be quite low in the field (cf. replication index ranking, https://replicationindex.com/2022/01/26/rr21/, last accessed on March 10, 2021).

Beyond current research practice, an additional contribution towards the development of open science practices in PNE can be made by *teaching staff* and *scientific supervisors*. Students at the earliest stage, particularly those enrolled in research programs, should be offered specific training on the implementation of open science practices, training on data and code sharing, and training on reporting standards in PNE to ensure the constant improvement of our field [[102], [103], [104]]. There are several resources that can help to incorporate open science practices in teaching environments (e.g., Framework for Open and Reproducible Research Training, FORRT, cf. https://forrt.org) and teachers and PIs are encouraged to incorporate this knowledge into their teaching and training.

## 5 Call for theory refinement and the development of methodological guidelines

In addition, beyond the more general strategies aimed at fostering sustainable change within a field, there are some PNE-specific issues that ought to be considered. This second important issue on the one hand concerns theory refinement (including rigorously testing auxiliary assumptions of common hypotheses, cf. call for action in Oxytocin research, [31]. On the other hand, and this is the aspect we want to focus on in this paper, it conveys the formulation and implementation of methodological guidelines, both concerning data assessment and analysis in PNE research. While it is essential to complement traditional incentive structures and publication schemes through the appreciation of open and transparent science practices, preregistration and RRs are not a universal solution for low replicability in a field [60]; they build upon good theories, methods and analyses and contribute to lay the ground for robust results that accelerate scientific process. Therefore, the comprehensive formulation of assessment and analysis guidelines facilitates informative reporting and increases inter-study comparability, which in turn enables replication efforts. Conceptual clarity and pre-specification of outcome and control variables may be especially critical to advance synthesis of PNE results, which is currently hampered by divergent operationalizations of key constructs (e.g., stress, allostatic load, different parameters of cortisol output, etc.). Consequently, calls for basic, methodological work and expert guidelines, as well as the control of the implementation thereof, should be raised and promoted more actively. The latter seems to be of utmost importance. A recent invited evaluation of the current state of the methodology for the assessment of the CAR revealed that although the respective expert consensus guidelines [33] have been highly cited, critical recommendations from these guidelines are only followed by a minority of researchers (Stalder et al., in prep). This is particularly noteworthy as the ‘guide for authors’ of the *Psychoneuroendocrinology* (PNEC) journal strongly recommends that these guidelines are followed. As a direct response to these unsatisfactory results, the PNEC editorial team are currently initiating a change to the reviewing procedure of research featuring CAR assessments, which will require authors to submit a filled in checklist on their adherence to the guidelines report criteria alongside with their article. This will foster transparency and assist reviewers' decision making by providing easily accessible information on the quality of respective data.

This provides an excellent example for the notion that an important role within this process is also likely to fall to journal editors and scientific societies who are in a position to enforce a firmer implementation of such best practice guidelines. Another important step could be the establishment of permanent task forces for the development of assessment and analysis guidelines. This way, the continued effort to create and update transparent best practices in PNE could be ensured. The issue to transparently report which of the guidelines were followed was recently addressed by the introduction of the Cortisol Assessment List (CoAL; [34], a tool to systematically document cortisol assessment in blood, urine and saliva. This approach can be seen as a first step to easily access information regarding data collection specifics, which could aid replication efforts. However, the field of PNE is more than just the collection of cortisol samples – further guidelines in other areas of endocrinological research are needed to improve our field sustainably. Ultimately, standardized reporting and the development of methodological guidelines are also crucial for the transfer of preclinical discoveries to evidence-based healthcare applications.

## 6 Conclusion & outlook

In summary, PNE has, in our opinion, still some ground to cover regarding the broad adoption of open science principles, but solutions exist, and their timely implementation could greatly aid in the advancement of the field. While the field of metascience is growing [15], PNE-specific work is scarce at this point. Simultaneously, more methodological work and the development of best practice guidelines to improve the reliability of our measurements are needed. Both will overall increase data quality and the reliability of findings, which benefits individual researchers as well as our field and, finally, society. As such, we expect that these steps will have a direct impact on PNE as a whole, and further on the translational value of our results. We (the authors of this article) are aware that this process takes time. We by no means want to imply that our own research reaches all benchmarks of open science, or indeed, that open science practices are flawless. However, we hope to initiate a collective effort within PNE that will over time benefit the whole field. Next to this overarching goal, we hope we could also highlight that there are indeed ‘selfish reasons’ for adopting open science practices that individual researchers can benefit from.

## Glossary

For more detailed information on these terms (and many more) see: https://forrt.org/glossary/,[1].

### 6.1 Open science

… an umbrella term that reflects the idea that “scientific knowledge of all kinds, where appropriate, should be openly accessible, transparent, rigorous, reproducible, replicable, accumulative, and inclusive, all which are considered fundamental features of the scientific endeavour” (cf [1]. Open science practices can refer to different aspects of research, e.g., data, methods and materials, analysis code, etc.

### 6.2 Preregistration

… describes the practice of registering a study's rationale and design as well as the hypotheses and analysis plans prior to data collection or analysis in a time-stamped document. Preregistration clearly separates confirmatory from exploratory data analyses.

### 6.3 Confirmatory data analysis

… refers to data analyses that were planned *a priori* and that are intended to test specific hypotheses.

### 6.4 Exploratory data analysis

… refers to data analyses that were not planned *a priori* but are conducted to discover and explore (unexpected) patterns in the data. Exploratory data analyses complement confirmatory analyses and can feed the formulation of new hypotheses.

### 6.5 Registered report

… is a publishing format that allows an in-principle acceptance (IPA) of a study design and analytical approach prior to the conductance of a study. Registered reports undergo a two-stage peer review: First, the study's rationale and methods are reviewed (and an IPA is decided if quality is high). Second, the complete manuscript including results and interpretation is reviewed after the conductance of the study. The study is published irrespective of the results (i.e., confirmation of hypotheses).

### 6.6 Reproducibility

… refers to the question: Can I arrive at the result of a study when using the *same* methods, materials, data, and analysis code (i.e., same sample, same material)? As such, it is the accuracy of results based on the *original* methods, materials, data, and analysis code and is sometimes also referred to as *computational reproducibility*. It can for example be tested by other researchers if data and analysis code of the original experiment are available.

### 6.7 Replicability

… refers to the question: Can I arrive at *similar* results of a study when using *comparable* methods, materials, analyses, and data? A result is replicable, if the repetition of the original experiment based on either *exactly* or *conceptually* comparable methods, materials, data, and analysis code yields the same conclusion. The term is used here to refer to both direct and conceptual replication.

A *direct replication* attempt exactly follows the original study protocol. If results of the original study are replicable, the originally reported effects can be shown repeatedly with the methods used and there is no reason to expect a different result in the replication attempt. A *conceptual replication* employs a different methodology (such as a different operationalization of independent or dependent variables) to test the original hypothesis in a different sample. Conceptual replications can thus provide evidence that the explanation for a finding is generalizable and not dependent on a certain methodology [106,107].

### 6.8 Secondary data analysis

… describes the reanalysis of data that were previously collected and analyzed in full or in part. Often, these analyses address research questions additional to the ones for which the data were initially collected.

Both registered reports and preregistration can be used to specify plans for secondary data analysis (‘secondary registration’ in the case of registered reports). This commonly involves a comprehensive and transparent summary of previous analyses and reflection of how they may have influenced new hypotheses. As registered reports are originally geared towards registration *prior* to data collection, not all registered report journals accept secondary registrations. For preregistration, specific templates exist for the preregistration of secondary data analysis.

## Supplemental material

Description of the screening process.

All articles published in PNEC and CPNEC in the year 2021 were screened by authors M.M., M.S., and L.P. as well as Lisa Haxel and Fiona Rambo. Articles were divided between these people, so that each volume was fully screened by only one person. Each article was screened for a set of keywords using the in-browser search function. Search terms were: prereg, regist, pre-reg, code, script, open data, avail, preprint, share, public, https, git, github, osf, arxiv, aspredicted, available, request. For all coded results, see: https://osf.io/pmk69/.

## CRediT author statement (alphabetical order)

Maria Meier: Conceptualization; Writing - Original Draft; Writing - Review & Editing; Methodology (of paper screening); Sebastian Laufer: Conceptualization; Writing - Original Draft; Writing - Review & Editing; Roman Linz: Conceptualization; Writing - Original Draft; Writing - Review & Editing; Methodology (of paper screening); Tina B. Lonsdorf: Conceptualization; Writing - Original Draft; Writing - Review & Editing; Sonia Lupien: Conceptualization; Writing - Original Draft; Writing - Review & Editing; Maurizio Sicorello: Methodology (of paper screening); Writing - Review & Editing; Tobias Stalder: Conceptualization; Writing - Original Draft; Writing - Review & Editing; Lara M.C. Puhlmann: Conceptualization; Writing - Original Draft; Writing - Review & Editing; Methodology (of paper screening)

## Conflict of interest

None.
